# A Generalized Chirp-Scaling Algorithm for Geosynchronous Orbit SAR Staring Observations

**DOI:** 10.3390/s17051058

**Published:** 2017-05-06

**Authors:** Caipin Li, Mingyi He

**Affiliations:** 1 School of Electronics and Information, Northwestern Polytechnical University, N 1, Dongxiang Rd, Xi’an 710129, China; licaipin2010@163.com; 2 China Academy of Space Technology (Xi’an), Weiqu Street, Xi’an 710100, China

**Keywords:** staring observation, geosynchronous SAR, fifth-order slant range mode, improved chirp-scaling algorithm

## Abstract

Geosynchronous Orbit Synthetic Aperture Radar (GEO SAR) has recently received increasing attention due to its ability of performing staring observations of ground targets. However, GEO SAR staring observation has an ultra-long integration time that conventional frequency domain algorithms cannot handle because of the inaccurately assumed slant range model and existing azimuth aliasing. To overcome this problem, this paper proposes an improved chirp-scaling algorithm that uses a fifth-order slant range model where considering the impact of the “stop and go” assumption to overcome the inaccuracy of the conventional slant model and a two-step processing method to remove azimuth aliasing. Furthermore, the expression of two-dimensional spectrum is deduced based on a series of reversion methods, leading to an improved chirp-scaling algorithm including a high-order-phase coupling function compensation, range and azimuth compression. The important innovations of this algorithm are implementation of a fifth-order order slant range model and removal of azimuth aliasing for GEO SAR staring observations. A simulation of an L-band GEO SAR with 1800 s integration time is used to demonstrate the validity and accuracy of this algorithm.

## 1. Introduction

Owing to its ability of all-weather and all-day Earth observation, synthetic aperture radar (SAR) has been widely applied in a variety of microwave remote sensing fields, such as disaster monitoring [1], topographic mapping [2], soil moisture estimation [3], oil spill observation [4], crop growth monitoring [5], forest cover measurement [6], wetland mapping [7], maritime surveillance [8,9,10], and so on. However, with increasing demands of wider imaging swath and shorter revisit period, it is difficult for low Earth orbit (LEO) SAR to satisfy the application requirements. To solve this problem, the Geosynchronous Orbit SAR (GEO SAR) concept has been put forward.

Compared with LEO SAR, GEO SAR not only has more advantages, such as wider swath, and shorter revisiting period [11,12,13,14,15,16], but it also can realize long time observation of ground targets even with staring, which will be beneficial to its applications in microwave remote sensing fields. Staring observation is the classical spotlight mode with a steering to a rotation center within the imaged scene [17]. GEO SAR’s staring observation can be obtained using a certain beam forming strategy that could be implemented by adjusting the antenna beam direction, or through satellite attitude steering [18].

The first challenge in GEO SAR staring observation image formation is the integration time which is extended to thousands of seconds in comparison with the hundreds of seconds in GEO SAR conventional observation using strip-map mode. In this case, conventional GEO SAR frequency domain algorithms, such as the modified chirp scaling (CS) algorithm [19,20], improved frequency domain focusing method [21,22], generalized Omega-K algorithm [23], and sub-aperture processing methods [24], cannot be directly used because of the inaccuracy of the corresponding slant models. In above algorithms, the slant range model based on the fourth model fails to precisely describe the range history of the ultra-long integration time in the staring observation. Zhao et al. [25] have proposed an improved RD imaging algorithm based on a fifth Doppler range model that is mainly used for strip-map SAR. Moreover, the Doppler range model in [25] does not consider the impact of “stop and go” assumptions. Yu et al. [26] have proposed a time-frequency scaling algorithm to correct the high-order azimuth variance in GEO SAR, but the imaging mode is still in strip-map mode. Conventional time domain processing algorithms, such as the back projection algorithm [27], are not restricted by the slant range model. However, due to the prohibitively heavy computation load and inefficient processing, they are not practical to use. Besides, in the thousand seconds of integration time, many factors would affect the imaging focus, such as atmospheric refractive index, orbit perturbation, etc. Several methods have been proposed to solve these problems. Ruiz et al. [28] proposed the method of retrieving the atmospheric phase screen (APS) and compensating its effects on GEO SAR focusing. To improve GEO SAR focusing influenced by perturbations, Dong et al. [29] recommended the use of accurate orbit measurements or some signal processing methods. The autofocus technique for phase error correction has been proven to be a robust technique that can compensate for the phase error caused by atmosphere or orbit perturbations [30]. Although the need for deeper understanding of the effect on GEO SAR imaging, this paper will focus on imaging processes. 

The other challenge encountered in GEO SAR staring observation of image formation is the azimuth aliasing as the spotlight SAR. Therefore, an imaging algorithm for GEO SAR staring observation should address the issues. This paper aims to study the main problems posed by above challenges.

The main contributions of this paper are as follows: first, a fifth-order slant range model considering the impact of the “stop and go” assumption is developed for GEO SAR staring observations. Then, an accurate 2-D spectral based on series inversions is deduced. And a two-step processing method is proposed to remove spectrum aliasing. At last, an improved chirp scaling algorithm based on fifth-order slant range is obtained to perform high-order-phase coupling function compensation, range and azimuth compression. 

This paper is organized as follows: the signal model based on the fifth-order slant range for GEO SAR is investigated in Section 2. In Section 3, a two-dimensional spectrum of the echo is derived. In Section 4, the imaging algorithm is deduced in detail and the algorithm flow chart is given. Section 5 gives the simulation results to validate the improved imaging algorithm. Finally, conclusions are summarized in Section 6.

## 2. Signal Model for GEO SAR Staring Observation

**Figure 1:**
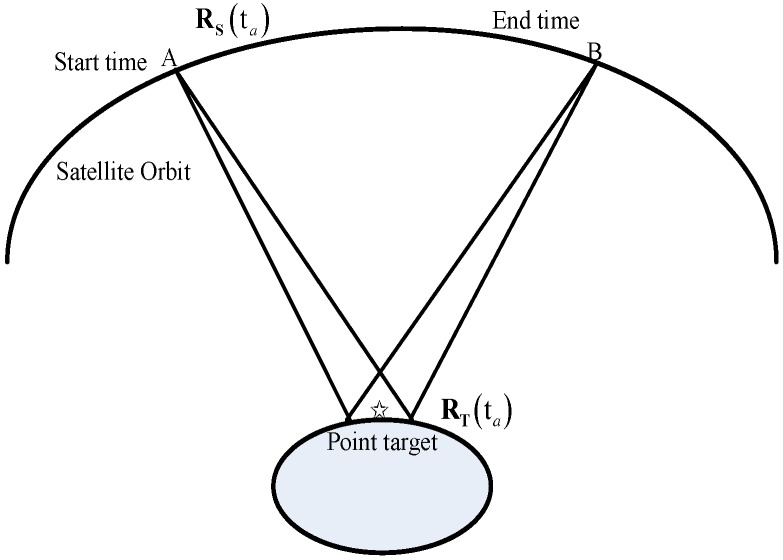
The geometry of satellite and ground targets.

The geometry of satellite and ground targets for GEO SAR staring observation is shown in Figure 1.

In the case of staring observations, the antenna (moving from position A to position B) is constantly steered to the point target which results in a long illumination time. Assuming the position vector of the satellite and the target vector in the Earth fixed coordinate system to be $R_{S}\left(t_{a}\right)$ and $R_{T}\left(t_{a}\right)$ respectively, and then the slant range from the satellite to the target point can be expressed as $R\left(t_{a}\right)=R_{S}\left(t_{a}\right)-R_{T}\left(t_{a}\right)$ , where $t_{a}$ is the azimuth time.

**Figure 2:**
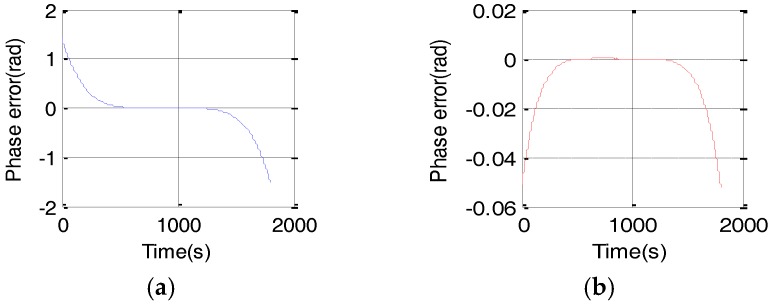
Phase errors caused by slant range mode. (**a**) Phase error of the fourth-order slant range model as a function of time and (**b**) Phase error of the fifth-order slant range model as a function of time.

**Figure 3:**
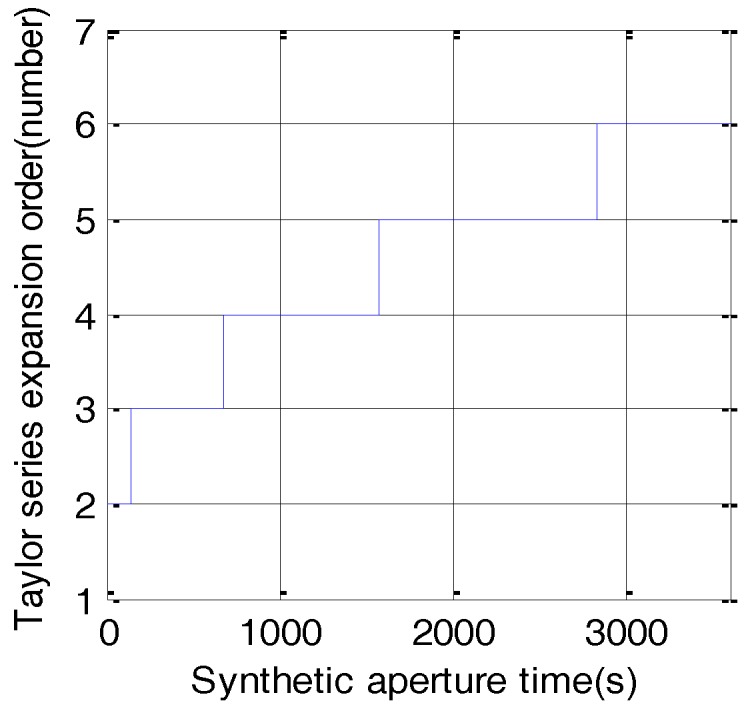
Relationship between different order slant range model and synthetic aperture time.

Taking a group of typical GEO SAR parameters, e.g., the semi-major axis of the orbit is 42,164 km and the orbit eccentricity is 0, the orbit inclination is 20° and the azimuth time is 1800s. From Figure 2, the maximal phase error caused by the conventional fourth-order slant range model is greater than π/4 whereas the fifth-order slant range model is far less than π/4. Nevertheless, the fourth-order slant range model cannot meet the requirement of the imaging algorithm. Consequently, the fifth-order range model is highly desired. Under the rule that phase error is less than π/4, we can get the relationship between different order slant range model and synthetic aperture time, which is shown in Figure 3.

As can be seen from the Figure 3, the third-order slant range model can be used when the synthetic aperture time does not exceed 680 s. The fourth-order slant distance model can be used when not more than 1580 s, and so on. However, when the synthetic aperture time exceeds 2830 s, the sixth-order slant range model must be used. In the 1800 s of synthetic aperture time, the fifth diagonal model can meet the requirements. Using Taylor series expansion in azimuth time, the slant range is extended to a fifth-order model by following equation:

(1) $$\left\|R\left(t_{a}\right)\right\|=R_{c}+V_{c}t_{a}+\frac{1}{2}A_{c}t_{a}^{2}+\frac{1}{6}B_{c}t_{a}^{3}+\frac{1}{24}C_{c}t_{a}^{4}+\frac{1}{120}D_{c}t_{a}^{5}+\cdots$$

where, the expression of $R_{c},\;V_{c},\;A_{c},\;B_{c},\;C_{c}$ can be obtained in [31]. The value of $D_{c}$ can be further expressed as:

(2) $$D_{c}=(15x_{1}^{5}/32-75x_{1}^{3}x_{2}/4+45x_{3}x_{1}^{2}/2+45x_{1}x_{2}^{2}/2-30x_{4}x_{1}-30x_{3}x_{2}+60x_{5})R_{ST}$$

in which:

$R_{ST}=\left|R_{ST}\right|$ ,

$$\begin{array}{l} x_{1}=2\frac{\langle R_{ST},V_{ST}\rangle }{{\left\|R_{ST}\right\|}^{2}},\\ x_{2}=\frac{\langle R_{ST},A_{ST}\rangle +\langle V_{ST},V_{ST}\rangle }{{\left\|R_{ST}\right\|}^{2}},\\ x_{3}=\frac{\langle R_{ST},B_{ST}\rangle /3+\langle V_{ST},A_{ST}\rangle }{{\left\|R_{ST}\right\|}^{2}},\\ x_{4}=\frac{\langle R_{ST},C_{ST}\rangle /12+\langle V_{ST},B_{ST}\rangle /3+\langle A_{ST},A_{ST}\rangle /4}{{\left\|R_{ST}\right\|}^{2}},\\ x_{5}=\frac{\langle R_{ST},D_{ST}\rangle /60+\langle V_{ST},C_{ST}\rangle /12+\langle A_{ST},B_{ST}\rangle /6}{{\left\|R_{ST}\right\|}^{2}},\\ R_{ST}=R_{S}-R_{T},\;V_{ST}=V_{S}-V_{T},\;A_{ST}=A_{S}-A_{T},\;B_{ST}=B_{S}-B_{T},\;C_{ST}=C_{S}-C_{T},\;D_{ST}=D_{S}-D_{T} \end{array}$$

where R, V, A, B, C are all state vectors and represent the satellite and target positions vector, velocity vector, acceleration vector, rate of the acceleration vector, and the rate of the acceleration vector, respectively. The subscript S and T denote position and target, respectively. Operator 〈,〉 represents the inner product operation. The expression of $D_{S}$ and $D_{T}$ can be obtained with the following equations:

(3) $$\begin{array}{ll} D_{S} & =\mu^{2}V_{S}/{\left|R_{S}\right|}^{6}-\;3\;\mu^{2}\langle R_{S},V_{S}\rangle R_{S}/{\left|R_{S}\right|}^{5}+\;9\;\mu A_{S}\langle R_{S},V_{S}\rangle /{\left|R_{S}\right|}^{5}\\ & +\;9\;\mu \langle V_{S},V_{S}\rangle V_{S}/{\left|R_{S}\right|}^{5}+\;9\;\mu \langle R_{S},A_{S}\rangle V_{S}/{\left|R_{S}\right|}^{5}+\;9\;\mu \langle V_{S},A_{S}\rangle R_{S}/{\left|R_{S}\right|}^{5}\\ & -\;45\mu {\langle R_{S},V_{S}\rangle }^{2}V_{S}/{\left|R_{S}\right|}^{7}-\;45\;\mu \langle R_{S},V_{S}\rangle \langle V_{S},R_{S}\rangle R_{S}/{\left|R_{S}\right|}^{7}\\ & -\;45\;\mu \langle A_{S},R_{S}\rangle \langle R_{S},V_{S}\rangle R_{S}/{\left|R_{S}\right|}^{7}-\;3\;\mu^{2}\langle R_{S},V_{S}\rangle R_{S}/{\left|R_{S}\right|}^{8}\\ & +\;9\;\mu^{2}\langle R_{S},V_{S}\rangle R_{S}/{\left|R_{S}\right|}^{8}+\;105\;\mu {\langle R_{S},V_{S}\rangle }^{3}R_{S}/{\left|R_{S}\right|}^{9} \end{array}$$

(4) $$D_{T}=W_{e}⊗C_{T}$$

where $W_{e}$ is angular velocity vector of the Earth rotation, μ is the constant of gravitation and operator ⊗ stands for the outer product. 

In the slant range, because of the ultra-long integration time, the effect of perturbing orbital elements on the satellite and target kinematic parameters should be taken into account [29]. As indicated in [31], the satellite and target kinematic parameters that can be obtained from the orbital elements include the following: a , i.e., the semimajor axis of the orbit, e , i.e., the eccentricity of the orbit, i , i.e., the inclination of the orbit, and *Ω*, i.e., the longitude of the ascending node, f , i.e., the true anomaly, w , i.e., the argument of perigee. Ignoring the slight stochastic perturbation errors, the osculating orbital elements can be expressed as:

(5) $$\{\begin{array}{c} a=a_{m}+{\left(\Delta a\right)}_{sec}+{\left(\Delta a\right)}_{lp}+{\left(\Delta a\right)}_{sp}\\ e=e_{m}+{\left(\Delta e\right)}_{sec}+{\left(\Delta e\right)}_{lp}+{\left(\Delta e\right)}_{sp}\\ i=i_{m}+{\left(\Delta i\right)}_{sec}+{\left(\Delta i\right)}_{lp}+{\left(\Delta i\right)}_{sp}\\ \Omega =\Omega_{m}+{\left(\Delta \Omega \right)}_{sec}+{\left(\Delta \Omega \right)}_{lp}+{\left(\Delta \Omega \right)}_{sp}\\ f=f_{m}+{\left(\Delta f\right)}_{sec}+{\left(\Delta f\right)}_{lp}+{\left(\Delta f\right)}_{sp}\\ w=w_{m}+{\left(\Delta w\right)}_{sec}+{\left(\Delta w\right)}_{lp}+{\left(\Delta w\right)}_{sp} \end{array}$$

where $a_{m},\;e_{m},\;i_{m},\;\Omega_{m},\;f_{m},\;w_{m}$ are the mean orbital elements; Δa, Δe, Δi, ΔΩ, Δf, Δw are the perturbing orbital elements; and the subscripts “sec”, “lp”, and “sp” denote the secular perturbation term, the longperiod term, and the short-period term, respectively. Therefore, by computation of the orbit perturbation parameters and the accurate measurement of the orbit, we can obtain the slant distance accurately.

In the conventional SAR echo signal model, the transmission point and receiving point can be considered to be at the same position. However, for GEO SAR, the orbit height is about 36,000 km, so this assumption is invalid [22]. To facilitate deduction and use of the algorithm, the impact of “stop-and-go” assumption must be compensated. Subsequently the imaging algorithm can be expressed in the form of monostatic SAR.

The error caused by the “stop-and-go” assumption on the slant range can be expressed as follows [22]:

(6) $$\Delta R\left(t_{a}\right)=\Delta R_{c}+\Delta V_{c}t_{a}+\frac{1}{2}\Delta A_{c}t_{a}^{2}+\frac{1}{6}\Delta B_{c}t_{a}^{3}+\cdots$$

where $\Delta R_{c}=\frac{\langle V_{ST},R_{ST}\rangle }{c}$ , $\Delta A_{c}=\frac{\langle B_{ST},R_{ST}\rangle +3\langle A_{ST},V_{ST}\rangle }{2c}$ , $\Delta B_{c}=\frac{\langle C_{ST},R_{ST}\rangle +4\langle B_{ST},V_{ST}\rangle }{6c}+\frac{\langle R_{ST},R_{ST}\rangle }{2c}$ , and c is the speed of light.

The reached range error using the same orbit parameters as in Section 2 and the third-order approximation is 0.416 mm, leading to a phase error far less than π/4. Thus, the error distance caused by the “stop and go” assumption can be fully derived from the third-order approximation. Taking the error caused by the “stop and go” assumption into account, the slant range in GEO SAR can be written as:

(7) $$R^{\prime }\left(t_{a}\right)\approx R_{c}^{\prime }+V_{c}^{\prime }\;t_{a}+\frac{1}{2}V_{c}^{\prime }t_{a}^{2}+\frac{1}{6}B_{c}^{\prime }t_{a}^{3}+\frac{1}{24}C_{c}t_{a}^{4}+\frac{1}{120}D_{c}t_{a}^{5}$$

where $R_{c}^{\prime }=R_{c}+\Delta R_{c}$ , $V_{c}^{\prime }=V_{c}+\Delta V_{c}$ , $A_{c}^{\prime }=A_{c}+\Delta A_{c}$ , $B_{c}^{\prime }=B_{c}+\Delta B_{c}$ .

According to the SAR principle, the echo signal after demodulation can be expressed as:

(8) $$s_{0}\left(t_{r},t_{a}\right)=w_{r}\left(t_{r}-\frac{2R^{\prime }\left(t_{a}\right)}{c}\right)w_{a}\left(t_{a}\right)\exp\left(-j\frac{4\pi f_{0}R^{\prime }\left(t_{a}\right)}{c}\right)\exp\left(j\pi K_{r}{\left(t_{r}-\frac{2R^{\prime }\left(t_{a}\right)}{c}\right)}^{2}\right)$$

where $t_{r}$ is the range time, $t_{a}$ is the azimuth time, $w_{r}$ and $w_{a}$ stand for range envelope and azimuth envelope, respectively; $f_{0}$ denotes carrier frequency, $K_{r}$ is the frequency modulate rate. $R^{\prime }\left(t_{a}\right)$ is the slant range depending on azimuth time.

## 3. Two-Dimensional Spectrum Analysis

The two-dimensional spectrum analysis plays an important role in frequency domain algorithms, and the improved imaging algorithm can be deduced from which. If the fast Fourier transform FFT is performed in the range domain, the echo signal can be written as:

(9) $$s_{1}\left(f_{r},t_{a}\right)=W_{r}\left(f_{r}\right)w_{a}\left(t_{a}\right)\exp\left(-j\frac{4\pi (f_{0}+f_{r})R^{\prime }\left(t_{a}\right)}{c}\right)\exp\left(-j\frac{\pi f_{r}^{2}}{K_{r}}\right)$$

where $f_{r}$ is the range frequency.

Then, we perform the azimuth FFT and using the stationary phase principle and series inversion principle. The signal’s two-dimensional frequency domain expression can be obtained as:

(10) $$\begin{array}{ll} \Theta \left(f_{r},f_{a}\right) & \approx -\pi \frac{f_{r}^{2}}{K_{r}}-\frac{4\pi \left(f_{c}+f_{r}\right)}{c}\{{R^{\prime }}_{c}-\frac{A_{1}}{2}{\left[-\frac{cf_{a}}{2\left(f_{c}+f_{r}\right)}-V_{c}^{\prime }\right]}^{2}\\ & -\frac{A_{2}}{3}{\left[-\frac{cf_{a}}{2\left(f_{c}+f_{r}\right)}-V_{c}^{\prime }\right]}^{3}-\frac{A_{3}}{4}{\left[-\frac{cf_{a}}{2\left(f_{c}+f_{r}\right)}-V_{c}^{\prime }\right]}^{4}-\frac{A_{4}}{5}{\left[-\frac{cf_{a}}{2\left(f_{c}+f_{r}\right)}-V_{c}^{\prime }\right]}^{5}\} \end{array}$$

where $f_{a}$ is azimuth frequency, and:

$$\{\begin{array}{c} A_{1}=\frac{1}{A_{c}^{\prime }}\\ A_{2}=-\frac{B_{c}^{\prime }}{{A_{c}^{\prime }}^{3}}\\ A_{3}=\frac{3{B_{c}^{\prime }}^{2}-A_{c}^{\prime }C_{c}}{6{A_{c}^{\prime }}^{5}}\\ A_{4}=-\frac{{B_{c}^{\prime }}^{3}}{2{A_{c}^{\prime }}^{7}}+\frac{7B_{c}^{\prime }C_{c}}{12{A_{c}^{\prime }}^{6}}-\frac{D_{c}}{{A_{c}^{\prime }}^{5}} \end{array}$$

because $\frac{f_{r}}{f_{c}}$ is far less than 1, so the series expansion can be represented in terms of ${\left(1+\frac{f_{r}}{f_{c}}\right)}^{-1}$ , ${\left(1+\frac{f_{r}}{f_{c}}\right)}^{-2}$ and ${\left(1+\frac{f_{r}}{f_{c}}\right)}^{-3}$ . With this substitution, the two-dimensional spectral phase can be rewritten as follows:

(11) $$\Theta \left(f_{r},f_{a}\right)\approx \varphi_{0}\left(f_{a}\right)+\varphi_{1}\left(f_{a}\right)f_{r}+\varphi_{2}\left(f_{a}\right){f_{r}}^{2}+\varphi_{3}\left(f_{a}\right){f_{r}}^{3}$$

where $\varphi_{0}\left(f_{a}\right)$ is the azimuth phase, which is irrelevant to range frequency, can be expressed as:

(12) $$\varphi_{0}\left(f_{a}\right)=-\frac{4\pi }{\lambda }[R_{c}^{\prime }+\frac{\lambda^{2}A_{1}}{8}{\left(f_{a}-f_{d}\right)}^{2}-\frac{\lambda^{3}A_{2}}{24}{\left(f_{a}-f_{d}\right)}^{3}+\frac{\lambda^{4}A_{3}}{64}{\left(f_{a}-f_{d}\right)}^{4}-\frac{\lambda^{5}A_{4}}{160}{\left(f_{a}-f_{d}\right)}^{5}]$$

where $f_{d}=-\frac{2V_{c}^{\prime }}{\lambda }$ , $\varphi_{1}\left(f_{a}\right)$ is the first order coupling phase describing RCM, which is relevant on range frequency can be expressed as:

(13) $$\begin{array}{ll} \varphi_{1}\left(f_{a}\right) & =-\frac{4\pi }{c}\{R_{c}+\frac{\lambda^{2}A_{1}}{8}\left[{\left(f_{a}-f_{d}\right)}^{2}+2f_{d}\left(f_{a}-f_{d}\right)\right]-\frac{\lambda^{3}A_{2}}{24}\left[2{\left(f_{a}-f_{d}\right)}^{3}+3f_{d}{\left(f_{a}-f_{d}\right)}^{2}\right]\\ & +\frac{\lambda^{4}A_{3}}{64}\left[3{\left(f_{a}-f_{d}\right)}^{4}+4f_{d}{\left(f_{a}-f_{d}\right)}^{3}\right]-\frac{\lambda^{5}A_{4}}{160}\left[4{\left(f_{a}-f_{d}\right)}^{5}+5f_{d}{\left(f_{a}-f_{d}\right)}^{4}\right]\} \end{array}$$

$\varphi_{2}\left(f_{a}\right)$ is the second-order coupling phase which contains the range modulation phase and the second-order cross-coupling, expressed as:

(14) $$\varphi_{2}\left(f_{a}\right)=\frac{\pi f_{a}^{2}}{f_{c}^{2}}\{\frac{\lambda A_{1}}{2}-\frac{\lambda^{2}A_{2}}{6}\left[3\left(f_{a}-f_{d}\right)\right]+\frac{\lambda^{3}A_{3}}{16}\left[6{\left(f_{a}-f_{d}\right)}^{2}\right]-\frac{\lambda^{4}A_{4}}{40}\left[10{\left(f_{a}-f_{d}\right)}^{3}\right]\}-\frac{\pi }{K_{r}}$$

$\varphi_{3}\left(f_{a}\right)$ is the third-order coupling phase which is relevant on the third- order range frequency. It can be defined in:

(15) $$\begin{array}{ll} \varphi_{3}\left(f_{a}\right) & =-\frac{\pi f_{a}^{2}}{f_{c}^{3}}\{\frac{\lambda A_{1}}{2}-\frac{\lambda^{2}A_{2}}{6}\left[4\left(f_{a}-f_{d}\right)+f_{d}\right]+\frac{\lambda^{3}A_{3}}{16}\left[10{\left(f_{a}-f_{d}\right)}^{2}+4f_{d}\left(f_{a}-f_{d}\right)\right]\\ & -\frac{\lambda^{4}A_{4}}{40}\left[20{\left(f_{a}-f_{d}\right)}^{2}+10f_{d}\left(f_{a}-f_{d}\right)\right]\} \end{array}$$

## 4. Imaging Algorithm

In this section, we concentrate on the corresponding imaging algorithm. The proposed algorithm includes two parts. The first part, the azimuth preprocessing, is used to remove the aliasing in the staring observation mode; the second one is the improved CS algorithm.

### 4.1. Azimuth Preprocessing

In the staring observation mode, the azimuth Doppler bandwidth is composed of two parts. One part is the beam coverage Doppler bandwidth of the radar, known as the instantaneous Doppler bandwidth as strip-map mode. The other part is steering of the beam around the observation point causing rotation of the Doppler center. It introduces an extra bandwidth called beam rotation bandwidth, which may be several times larger than the instantaneous Doppler bandwidth. In space-borne SAR, to obtain a larger imaging swath, pulse repetition frequency is usually designed by instantaneous Doppler bandwidth that will cause the azimuth spectrum aliasing in the staring imaging mode. To remove the aliasing, two methods are usually adopted. One is the sub-aperture method [32,33], where the data is divided into small segments in the azimuth and imaging respectively processing. However, the sub-aperture operation is inefficient and complex. The other one is azimuth preprocessing based on two-step. The two step processing method is illustrated in [34,35,36], usually used to remove the aliasing in spotlight SAR and sliding-spotlight SAR. We employ it to remove azimuth aliasing in staring observation. Subsequently, the signal aliasing is removed, resulting in imaging processing without ambiguity.

### 4.2. Improved CS Algorithm

Due to the curved trajectory and high order slant mode, classical imaging algorithms cannot be directly used in the case of GEO SAR. Therefore, an improved CS algorithm based on the fifth-order slant range is adapted to imaging processing. After the azimuth preprocessing, the signal third-order coupling phase can be compensated in frequency domain. The reference function is described as:

(16) $$H_{1}\left(f_{r},f_{a},R_{ref}\right)=\exp\left(j\varphi_{3}\left(f_{a},R_{ref}\right){f_{r}}^{3}\right)$$

where $R_{ref}$ is the slant range in the beam center.

Then, the range inverse fast Fourier transform (IFFT) of the signal is computed, and the expression can be obtained through the following expression:

(17) $$S\left(t_{r},f_{a};R_{c}^{\prime }\right)=W_{r}\left(t_{r}+\frac{\varphi_{1}\left(f_{a}\right)}{\pi }\right)W_{a}\left(f_{a}-f_{d}\right)\exp\left(j\varphi_{0}\left(f_{a};R_{c}^{\prime }\right)-j\frac{\pi^{2}}{\varphi_{2}\left(f_{a}\right)}{\left(t_{r}-t_{d}\left(f_{a},R_{c}^{\prime }\right)\right)}^{2}\right)$$

where, $t_{d}\left(f_{a},R_{c}^{\prime }\right)=-\varphi_{1}\left(f_{a},R_{c}^{\prime }\right)/(2\pi )$ .

The next step is to determine the scaling factor of the improved CS algorithm. Due to the high-order slant mode, the linear scaling factor is not easy to get and difficult to describe with the indicated equation. However, we can get it under the numerical polynomial fitting model as described in [37].

According to the numerical fitting, the linear scaling factor $C_{s}\left(f_{a}\right)$ can be obtained, and the line module frequency scaling function is described as:

(18) $$H_{2}\left(t_{r},f_{a},R_{ref}\right)=\exp\left(j\pi \left(C_{s}\left(f_{a}\right)-1\right)\frac{\pi }{\varphi_{2}\left(f_{a},R_{ref}\right)}{\left(t_{r}-t_{d}\left(f_{a},R_{ref}\right)\right)}^{2}\right)$$

After multiplying by the linear scaling function and computing the ranged FFT, the expression of the echo signals can be expressed as follows:

(19) $$\begin{array}{r} S\left(t_{r},f_{a}^{\prime };R_{c}^{\prime }\right)=W_{r}\left(-\frac{\pi f_{r}}{\varphi_{2}\left(f_{a}^{\prime },R_{c}\right)C_{s}\left(f_{a}\right)}\right)W_{a}\left(f_{a}-f_{d}\right)\exp\left(-\frac{j\varphi_{2}\left(f_{a},R_{c}^{\prime }\right)f_{r}^{2}}{C_{s}\left(f_{a}\right)}\right)\\ \exp\left(-j\frac{4\pi f_{r}}{c}\left(R_{c}^{\prime }+R_{ref}\left(C\left(f_{a}\right)-1\right)\right)\right)\exp\left(-j\varphi_{0}\left(f_{a},R_{c}^{\prime }\right)\right)\\ \exp\left(-j\frac{4\pi^{2}}{c^{2}}C_{s}\left(f_{a}\right)\left(C_{s}\left(f_{a}\right)-1\right)\frac{1}{\varphi_{2}\left(f_{a}^{\prime };R_{c}^{\prime }\right)}{\left(R_{c}^{\prime }-R_{ref}\right)}^{2}\right) \end{array}$$

Then, the phase of the range cell migration correction phase and the secondary range compression phase are compensated in the 2-D frequency domain. The function can be expressed as:

(20) $$H_{3}\left(t_{r},f_{a},R_{ref}\right)=\exp\left(j\pi \frac{f_{r}^{2}}{Cs(f_{a})\varphi_{2}\left(f_{a},R_{ref}\right)}\right)\exp\left(j\frac{4\pi \left(C_{s}\left(f_{a}\right)-1\right)R_{ref}}{c}\right)$$

After applying range IFFT, the azimuth compression and residual phase functions are presented by a fifth-order polynomial in the frequency domain, which is given by:

(21) $$H_{4}\left(t_{r},f_{a},R_{ref}\right)=\exp\left(j\varphi_{0}\left(f_{a}\right)\right)\exp\left(j\frac{4\pi^{2}}{c^{2}}\frac{\left(C_{s}\left(f_{a}\right)-1\right)C_{s}\left(f_{a}\right)}{\varphi_{2}\left(f_{a},R_{ref}\right)}{\left(R_{c}^{\prime }-R_{ref}\right)}^{2}\right)$$

**Figure 4:**
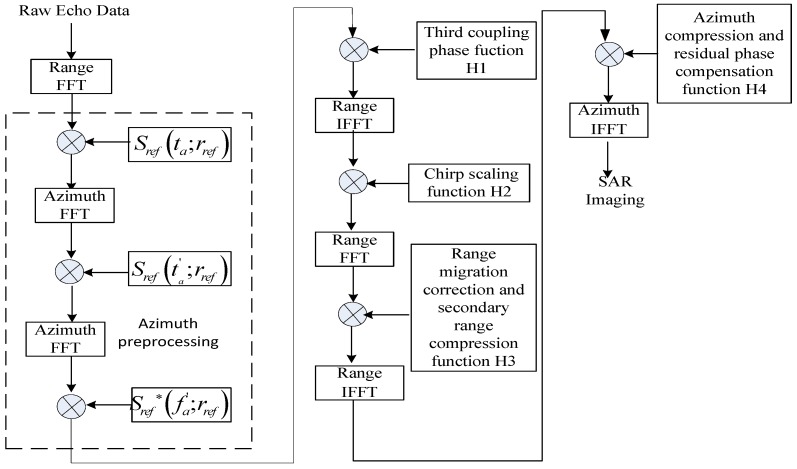
Flowchart of the imaging algorithm.

Finally, the echo data are transformed into the 2-D time domain, and the image is obtained. The flowchart of the imaging algorithm is shown in Figure 4.

## 5. Simulation Results

In order to validate the algorithm, simulation experiments are performed. The imaging quality of the proposed algorithm is evaluated. The parameters used in our experiments are described in Table 1.

**Figure 5:**
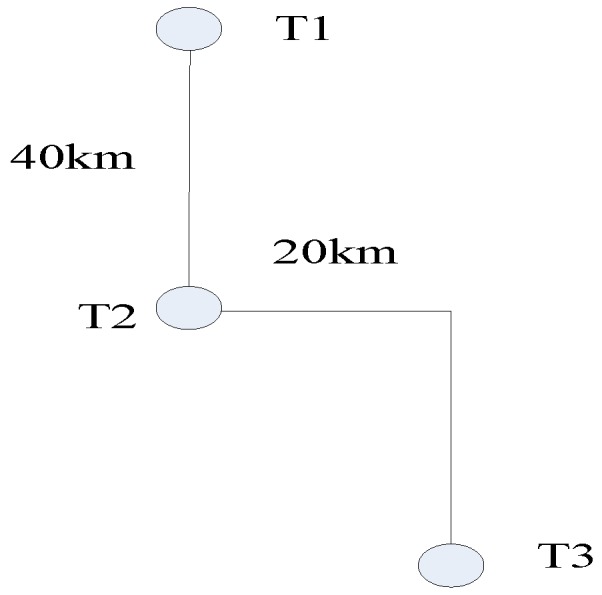
Target positions in the scene.

The satellite orbit time is selected at perigee passage time 0 and the designed imaging scene with size of 80 km × 40 km is shown in Figure 5. The integration time is 1800 s for the targets in the scene.

**Figure 6:**
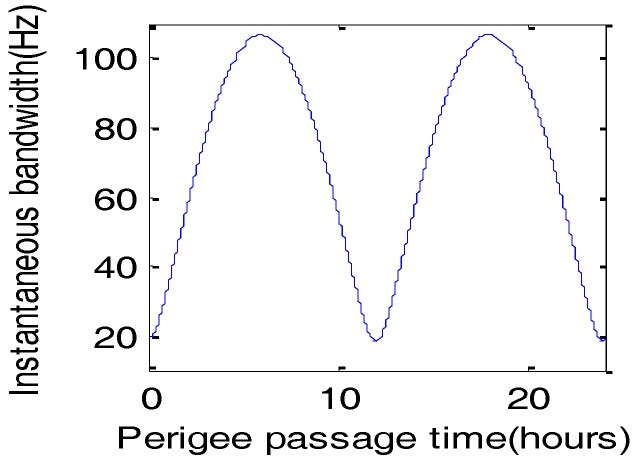
The instantaneous bandwidth.

The target T2’s scene bandwidth including the extra bandwidth introduced by the steering of the antenna and the instantaneous bandwidth of the beam is calculated, which is about 260 Hz. The instantaneous bandwidth is varied with the perigee passage time, as shown in Figure 6. It can be seen that the instantaneous bandwidth is about 20 Hz at perigee passage time 0 h, far less than scene bandwidth.

**Figure 7:**
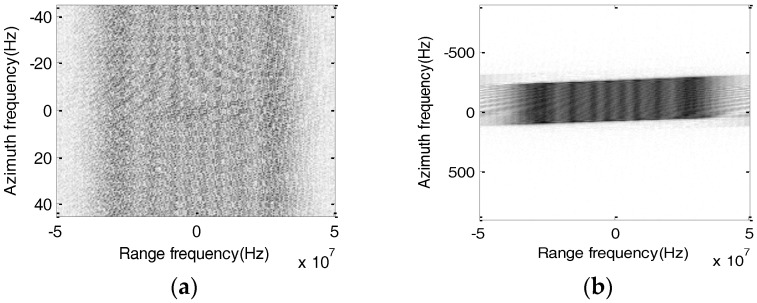
Signal spectrum without and with azimuth pre-processing. (**a**) signal spectrum aliasing without azimuth pre-processing;(**b**) signal spectrum aliasing removed with azimuth pre-processing.

The pulse repetition frequency is selected to 90 Hz. Figure 7a shows that the aliasing is occurs in the echo spectrum. However, when the operation of the azimuth preprocessing is used, the aliasing in the azimuth is removed, as shown in Figure 7b.

### 5.1. Simulation Results of the Conventional Algorithm

**Figure 8:**
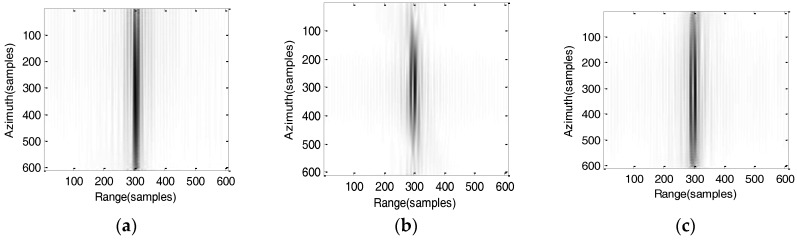

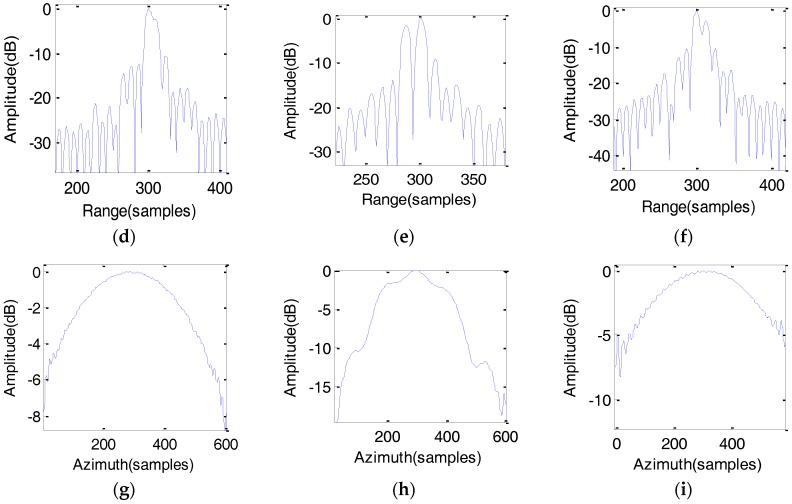
Imaging results by using conventional fourth-order algorithm (**a**) Target T1; (**b**) Target T2; (**c**) Target T3; (**d**) Range profile of point target T1; (**e**) Range profile of point target T2; (**f**) Range profile of point target T3; (**g**) Azimuth profile of point target T1; (**h**) Azimuth profile of point target T2; (**i**) Azimuth profile of point target T3.

To compare the imaging results of algorithm, the conventional algorithm based on the fourth-order slant range mode is used [19]. The imaging results are shown in Figure 8. It can be clearly seen that all the point targets are severely defocused in the scene.

### 5.2. Simulation Results of the Improved Algorithm

**Figure 9:**
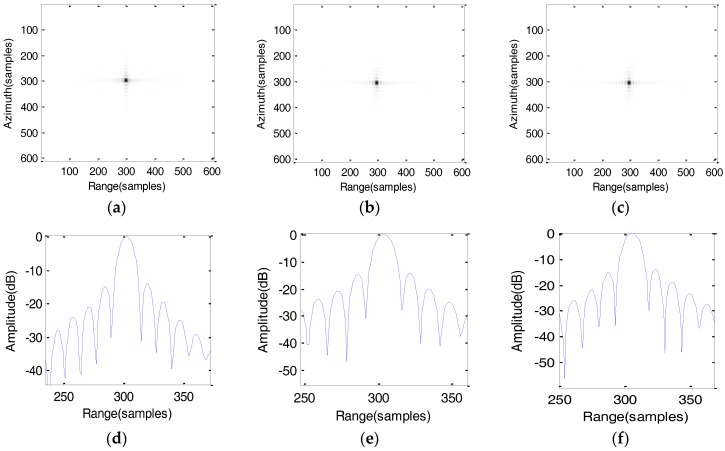

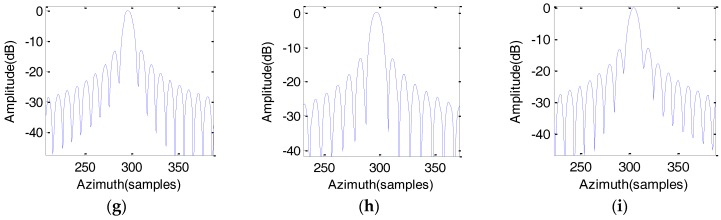
Imaging results at perigee passage time 0 h. (**a**) 2-D contour of point target T1; (**b**) 2-D contour of point target T2; (**c**) 2-D contour of point target T3; (**d**) Range profile of point target T1; (**e**) Range profile of point target T2; (**f**) Range profile of point target T3; (**g**) Azimuth profile of point target T1; (**h**) Azimuth profile of point target T2; (**i**) Azimuth profile of point target T3.

The improved scaling imaging algorithm based on the fifth-order slant model is used for imaging processing. The imaging results are shown in Figure 9.

The imaging performance including resolution (RZ), peak sidelobe rate (PSLR) and integrated sidelobe ratio (ISLR) of point targets are evaluated without windowing treatment as shown in Table 2. In order to compare the imaging results obtained by proposed algorithm and algorithm in [19], simulation results of the algorithm in [19] are also shown in Table 2.

Comparing the analytical results utilizing the improved algorithm of T1, T2 and T3, the PSLR in azimuth slightly decreases along the azimuth direction, which is mainly caused by the azimuth-variant compressed function. Similarly, the PLSR and ISLR in range are also slowly falling along the range direction which is mainly caused by the differential RCMC in range for the degradation, whereas from the table can be seen clearly that imaging performance close to the theoretical value which can meet the demand of imaging. However, azimuth and range defocusing occurs by using the algorithm [19], therefore, the azimuth resolution and ISLR has seriously deteriorated. By comparison, the proposed algorithm has better performance.

**Figure 10:**
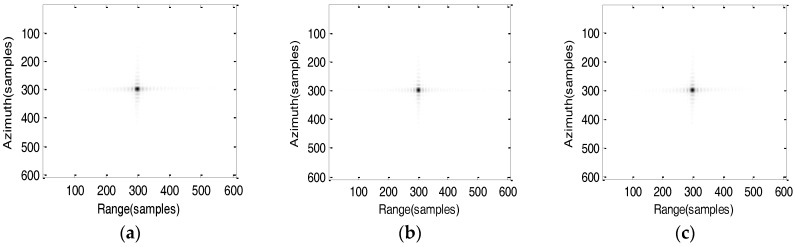

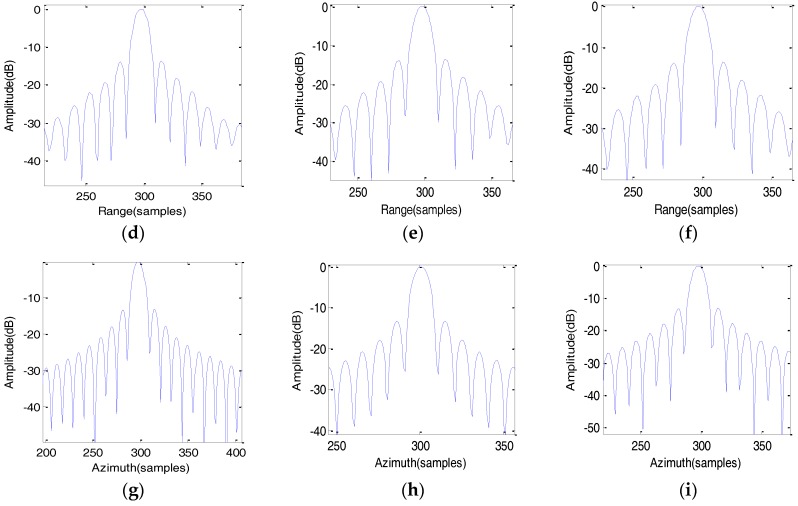
Imaging results at perigee passage time 2 h. (**a**) 2-D contour of point target T1; (**b**) 2-D contour of point target T2; (**c**) 2-D contour of point target T3; (**d**) Range profile of point target T1; (**e**) Range profile of point target T2; (**f**) Range profile of point target T3; (**g**) Azimuth profile of point target T1; (**h**) Azimuth profile of point target T2; (**i**) Azimuth profile of point target T3.

In order to further demonstrate the advantage of the proposed algorithm, different orbital positions such as at perigee passage time 2 h are randomly selected for imaging simulation. In the case of satellite yaw angle control, the imaging results obtained are shown in Figure 10.

The imaging performance at perigee passage time 2 h are evaluated without windowing treatment as shown in Table 3. As shown in Table 3, the resolution is slightly different from Table 2, which is caused by the orbital characteristics. The ideal PSLR should be −13.26 dB. The maximal loss of range and azimuth PSLRs is less than 0.23 dB, indicating good focusing quality. The range and azimuth ISLRs over the whole swath are around the standard value, also indicating good focusing quality.

In order to further verify the improved algorithm, the real image is used (Huashan Mountain, Shaanxi, China, obtained from sliding rail vehicle experiment) as the input radar cross section information in echo generation. The simulation parameters are the same as listed in Table 1. The synthetic aperture time is 1800 s. Both the conventional algorithm and the improved algorithm are used for focusing the SAR raw data. The imaging results of Huashan Mountain area are depicted.

**Figure 11:**
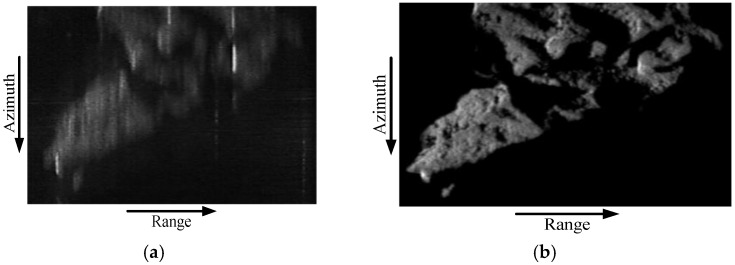
Comparison of imaging results of Huashan Mountain area. (**a**) conventional algorithm; (**b**) improved algorithm based on the fifth-order slant model.

As can be seen from Figure 11, it is obvious that the well-focused results are obtained by the improved scaling imaging algorithm based on the fifth-order slant model and as shown in Figure 11b, but the conventional algorithm results are defocusing as shown in Figure 11a. All the point target simulation results and real data simulation results prove the effectiveness of the improved algorithm.

## 6. Conclusions

GEO SAR has recently received increasing attention because of its great potential for future spaceborne SAR missions. An imaging algorithm for GEO SAR with staring observation is proposed. Based on the fifth-order slant range mode, the 2-D spectrum is deduced with consideration of the impact of “stop-and-go” assumption, and then a two step processing method is used to remove the signal spectrum aliasing. An improved scaling algorithm is presented for imaging processing. Finally, based on the orbit parameters and radar parameters, simulation experiments have demonstrated that the algorithm is effective for an L-band GEO SAR with 1800 s integration time. Besides, when it comes to longer synthetic aperture and wider swaths, the effects of spatial variation, atmospheric refractive index and orbit perturbation on image formation and the higher order slant model should be studied in depth. All of these will be major topics in our future work. 

## Figures and Tables

**Table 1:** Simulation Parameters.

Parameters	Value
Orbit semi-major axis	42,164 km
Orbit eccentricity	0
Orbit inclination	20°
Argument of perigee	95°
Right ascension of ascending node	97°
Radar frequency	1.25 GHz
Pulse repetition frequency	90 Hz
Bandwidth	80 MHz
Antenna beam width	0.5°
Off-nadir angle	3.5°

**Table 2:** Imaging results of point targets at perigee passage time 0 h.

Target Positions	Proposed Algorithm						Algorithm in [19]					
	Range			Azimuth			Range			Azimuth		
	RZ (m)	PSLR (dB)	ISLR (dB)	RZ (m)	PSLR (dB)	ISLR (dB)	RZ (m)	PSLR (dB)	ISLR (dB)	RZ (m)	PSLR (dB)	ISLR (dB)
T1	3.80	−13.22	−9.83	2.11	−13.02	−9.35	4.75	−2.357	−7.35	46	−4.21	−2.52
T2	3.86	−13.27	−9.75	2.02	−13.18	−9.68	3.81	−2.865	8.86	32	−2.36	1.25
T3	3.92	−13.20	−9.91	2.13	−13.05	−9.57	4.63	−1.08	−1.08	48	−5.01	−1.63

**Table 3:** Imaging results of point targets at perigee passage time 2 h.

Target Positions	Range			Azimuth		
	RZ (m)	PSLR (dB)	ISLR (dB)	RZ (m)	PSLR (dB)	ISLR (dB)
T1	3.82	−13.08	−9.52	2.35	−13.20	−9.45
T2	3.93	−13.19	−9.69	2.27	−13.16	−9.71
T3	4.01	−13.06	−9.65	2.47	−13.03	−9.35
